# Engineering herbicide‐resistant oilseed rape by CRISPR/Cas9‐mediated cytosine base‐editing

**DOI:** 10.1111/pbi.13368

**Published:** 2020-03-10

**Authors:** Jian Wu, Chen Chen, Guiyu Xian, Dongxiao Liu, Li Lin, Shengliang Yin, Qinfu Sun, Yujie Fang, Hui Zhang, Youping Wang

**Affiliations:** ^1^ Key Laboratory of Plant Functional Genomics of the Ministry of Education Yangzhou University Yangzhou China; ^2^ Jiangsu Key Laboratory of Crop Genomics and Molecular Breeding Yangzhou University Yangzhou China; ^3^ College of Life Science Shanghai Normal University Shanghai China

**Keywords:** *Brassica napus*, *ALS*, *AHAS*, *base*‐editing, *CRISPR*, *herbicide* resistant

Base editor technology that enables precise base‐editing has been developed by employing Cas9 nickase (nCas9) or deactivated Cas9 (dCas9) fused to an enzyme with base conversion activity, named cytidine‐deaminase‐mediated base editor (CBE, C•G to T•A) or adenine‐deaminase‐mediated base editor (ABE, A•T to G•C) (Gaudelli *et al.*, 2017; Komor *et al.*, 2016). The base editor systems have been successfully applied in several plant species, including *Arabidopsis*, rice, wheat, maize, tomato and cotton (Mao *et* *al.*, 2019). However, it is not clear whether base editing will work in allotetraploid oilseed rape (*Brassica napus*), one of the world’s most important oil crops. As weeds are a major threat to oilseed rape production, cultivation of herbicide‐tolerant varieties is the most cost‐effective tool to manage weeds. Acetolactate synthase (ALS), a key enzyme for the biosynthesis of branched‐chain amino acids, is the target site of several important herbicides (Powles and Yu, 2010). ALS harboring point mutations could confer sufficient tolerance to herbicidal ALS inhibitors. In this study, we report an efficient CBE system that was employed to create herbicide‐resistant *B. napus* by generating a precisely edited *BnALS* genes.

Based on the released *B. napus* genome information, cultivars Darmor‐bzh (Chalhoub *et al.*, 2014) and ZS11 (Sun *et al.*, 2017) both contain five *BnALS* copies (Figure 1a). Phylogenetic analysis was performed to reveal the evolutionary relationships among the *BnALS* copies. It showed that *BnALS1* and *BnALS3* are highly conserved, exhibiting 92.8% amino acid sequence homology with *Arabidopsis ALS* (Figure 1a). In contrast, *BnALS2*, *BnALS4* and *BnALS5* were distantly related to *AtALS*, suggesting that they may have undergone subfunctionalization after gene duplication (Figure 1a). Transcriptome data indicated that *BnALS1* and *BnALS3* were constitutively expressed in all tissues of *B. napus*, while *BnALS4* and *BnALS5* were not expressed in all tissues. *BnALS2* was expressed only in the ovary and in the seeds at 14 days after flowering (Figure 1b). Taken together, the above results indicate that *BnALS1* and *BnALS3* are likely have essential ALS housekeeping functions, as indicated in previous studies (Ouellet *et al.*, 1992); this suggests that *BnALS1* and *BnALS3* are ideal herbicide resistance targets for base editing in *B. napus*.

**Figure 1:**
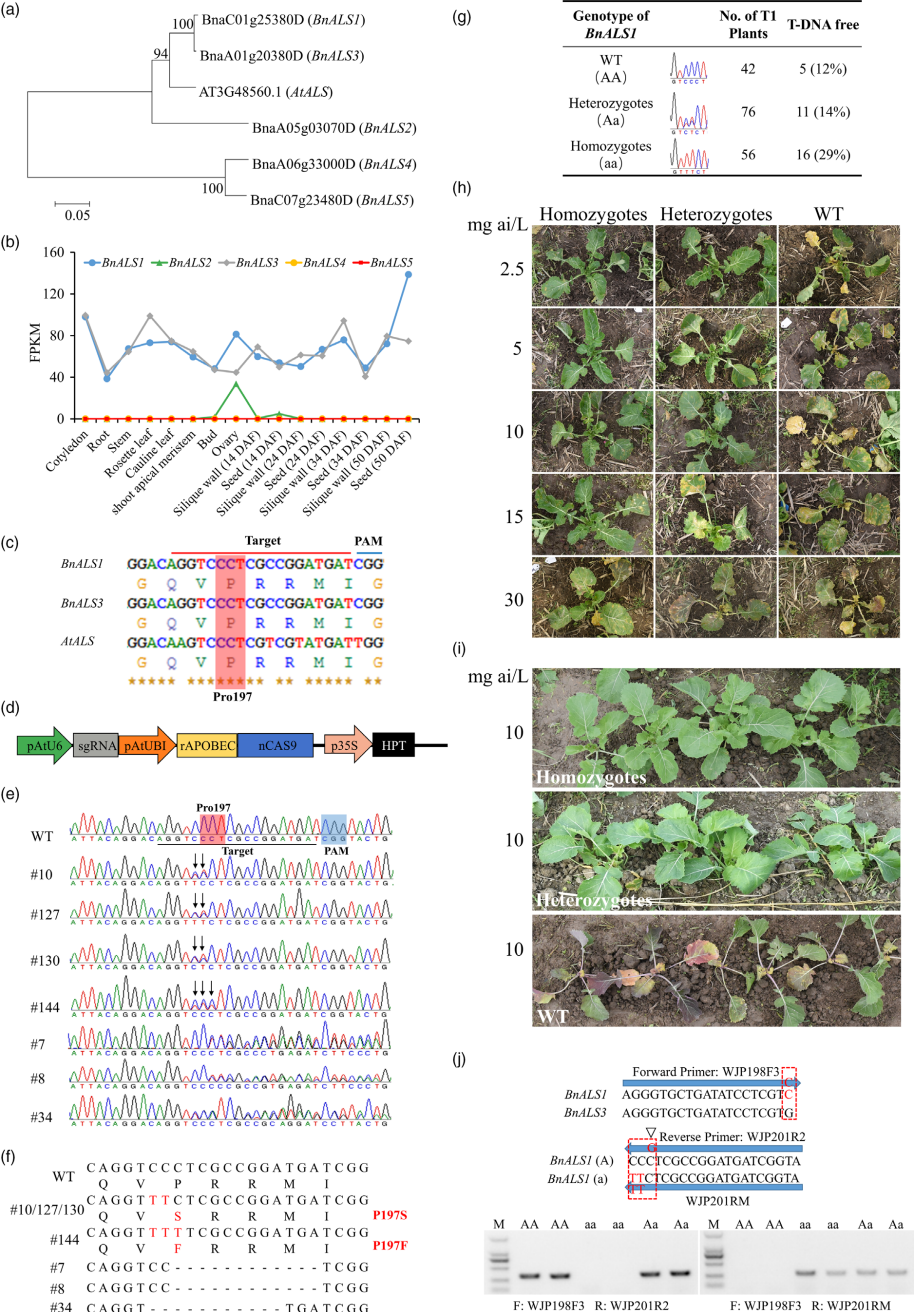
Creation of herbicide‐resistant plants by the CRISPR/Cas9‐mediated base‐editing system in *B. napus*. (a) Phylogenetic analysis of *ALS* homologs in *B. napus* and *Arabidopsis*. (b) Expression patterns of *BnALS* genes in diverse tissues of *B. napus* line J9712 determined by transcriptome sequencing. DAF, days after flowering. FPKM, fragments per kilobase of transcript per million fragments mapped. (c) The target sites of *BnALS1* and *BnALS3*. (d) Schematic of the cytidine base‐editing vector. (e) Sequence chromatograms of 7 T0 plants with editing events. Arrows point to the positions with edited bases. (f) The sequences of edited plants. Substitutions and deletions are indicated in red font and hyphens, respectively. (g) The segregation pattern of *BnALS1* in the T1 generation of the #10 plant according to the Sanger sequencing results. (h, i) Symptoms of wild‐type plants and P197S mutants 20 days after treatment with tribenuron‐methyl. (j) Allele‐specific markers were developed to discriminate among the WT, heterozygous and homozygous mutants. The 3’ ends of each primer correspond to one or two single‐nucleotide polymorphisms, which are indicated in red. The positions of the additional mismatched bases in the reverse primer WJP201R2 are marked with triangles.

Amino acid substitution at position P197 (numbered according to the corresponding sequence of *A. thaliana*) endows various plants with resistance to herbicides, such as tribenuron‐methyl (Chen *et al.*, 2017). We therefore selected this region as the target site for creating herbicide‐resistant oilseed rape by converting C to T at codon P197 of both *BnALS1* and *BnALS3* using a CBE system (Figure 1c). This sgRNA also targets *BnALS2.* The CBE vector used in this study contains a hygromycin phosphotransferase (HPT) selection marker, sgRNA transcription, rat cytidine deaminase (rAPOBEC1), D10A Cas9 nickase (nCas9) and a uracil glycosylase inhibitor (UGI) (Figure 1d). sgRNA transcription was driven by the *Arabidopsis* U6 promoter, and the cassette rAPOBEC1‐nCas9‐UGI was amplified from the pnCas9‐PBE plasmid (Addgene: #98164) and driven by the *Arabidopsis* ubiquitin 1 promoter.

The construct was transformed into the pure *B. napus* line J9712 using the *Agrobacterium tumefaciens*‐mediated hypocotyl method, generating 230 independent T0 plants. From PCR analysis using vector‐specific primers, 217 (94.3%) independent plants were positive transformants harboring T‐DNA insertions. We amplified the target regions of *BnALS1*‐*3* by PCR using gene‐specific primers and subjected the PCR products to Sanger sequencing. Based on the sequencing results, 7 (3.2%) of the 217 T0 plants exhibited editing events that produced superimposed sequencing chromatograms in the target site of *BnALS1* (Figure 1e). However, we could not find any mutations in the target region of *BnALS2* or *BnALS3*. For the *BnALS1* target, four plants (#10, #127, #130 and #144) had C–T substitutions at positions 5‐7 of the protospacer (scoring PAM as position 21‐23); the remaining three plants (#7, #8 and #34) had indels (Figure 1e). Plants #10, #127 and #130 had two C–T substitutions in the target site, but only one edited allele encoded a different amino acid (P197S, Figure 1f). Plant #144 harbored three C–T substitutions in the target site, resulting in a P197F amino acid substitution (Figure 1f). TA cloning and sequencing results further confirmed the editing events, showing that #7, #8 and #34 carried 12, 12 and 11 base pair deletions, respectively (Figure 1e). Both the sequencing chromatograms of the PCR product and the TA cloning results indicated that all mutant lines were heterozygous (Figure 1e,f). The base‐editing efficiency for *BnALS1* was approximately 1.8% (4/217).

To evaluate the possible off‐target potential in the present study, a total of 14 putative off‐target sites were predicted using the CRISPR‐P version 2.0 program (Liu *et al.*, 2017). Sequencing of all these predicted potential off‐target sites for the four base‐editing mutations did not reveal any editing events.

To obtain stable homozygous mutants and test whether the base‐editing mutants are inherited, plant #10 were self‐pollinated, and individual T1 progeny were genotyped via sequencing of the PCR products of the *BnALS1* target site. A total of 42 wild‐type plants (WT), 76 heterozygous mutants and 56 homozygous mutants were detected in the T1 progeny (Figure 1g), which indicated that the allelic mutation was successfully transmitted to the T1 generation, with an expected 1:2:1 monogenic segregation pattern (χ^2^ = 5.03, *P *> 0.05). Moreover, 16 homozygous mutants without exogenous T‐DNA could be obtained in the T1 generation (Figure 1g). However, we still could not find any new editing events in the target region of *BnALS2* and *BnALS3* in the T1 generation.

To determine whether the base‐editing mutants can confer oilseed rape with herbicide resistance, P197S mutants and WT (3 plants per genotype) were sprayed with various concentration of tribenuron‐methyl (Ryan Pingan, Henan, China, 20 mL/plant) at the 5‐6 leaf stage. Twenty days after the treatment, WT was severely damaged, even at a very low concentration (2.5 mg ai/L), whereas the heterozygous and homozygous mutants showed no symptoms of herbicide injury when the dosages were increased to 10 and 15 mg ai/L, respectively (Figure 1h). Strikingly, the homozygotes can even survive at the dosage of 30 mg ai/L, displaying a higher level of herbicide resistance than the heterozygotes (Figure 1h). Further spraying 10 mg ai/L tribenuron‐methyl (three times the field‐recommended dose) on more plants confirmed the herbicide resistance of the P197S mutants (Figure 1i).

In addition, to easily discriminate between the WT and P197S mutant alleles, allele‐specific PCR markers were developed. The forward primer was *BnALS1* specific and could distinguish between *BnALS1* and other homologous genes. While the target site was chosen to design the reverse primers, the 3’ end corresponded to the two base‐editing sites (Figure 1j). Moreover, one additional mismatched base was introduced into the reverse primer for the WT genotype immediately in front of the two SNP sites to reduce nonspecific amplification (Figure 1j). Thus, both gene specificity and allele specificity were successfully obtained, and the approach was sufficiently robust to easily discriminate among the WT, heterozygous and homozygous mutants (Figure 1j).

In summary, we successfully established a base‐editing system that can efficiently introduce C to T conversion in sgRNA targets in oilseed rape. The CBE would be a very useful tool for gene function studies and precision molecular breeding in oilseed rape. Moreover, the *BnALS*1 gene was precisely edited at position P197 by CBE system, conferring tribenuron‐methyl resistance to oilseed rape. The P197S substitution in *BnALS*1 generates a novel herbicide‐resistant mutant in oilseed rape, and the transgene‐free homozygous mutant with its allele‐specific markers can be used for breeding herbicide resistance and thus might facilitate weed management in oilseed rape production.

## Conflict of interest

The authors declare no competing interests.

## Author contributions

Y.W. and H.Z. conceived the study; J.W., C.C., G.X., D.L., L.L. and S.Y. performed the experiments; J.W. wrote the manuscript; Q.S. and Y.F. revised the manuscript.
